# Hypersensitivity pneumonitis associated with home mold exposure: A retrospective cohort analysis

**DOI:** 10.1371/journal.pone.0323093

**Published:** 2025-05-08

**Authors:** Traci N. Adams, Carrie A. Redlich, Craig S. Glazer, Mridu Gulati

**Affiliations:** 1 Division of Pulmonary and Critical Care Medicine, University of Texas Southwestern Medical Center, Dallas, Texas, United States of America; 2 Yale Occupational Medicine and Environmental Medicine, Yale School of Medicine, New Haven, Connecticut, United States of America; 3 Section of Pulmonary, Critical Care and Sleep Medicine, Yale School of Medicine, New Haven, Connecticut, United States of America; Kurume University School of Medicine: Kurume Daigaku Igakubu Daigakuin Igaku Kenkyuka, JAPAN

## Abstract

**Background:**

Home mold exposure is a commonly overlooked cause of hypersensitivity pneumonitis. This is in part due to the limited literature supporting the association as well as the lack of exposure characterization available in reported cases. Notably, climate change, extreme weather patterns and frequent flooding continue to create conditions that promote home mold growth. As remediation has the potential to improve outcomes, clinicians need to remain vigilant in searching for and identifying potential mold exposure in suspected HP patients. The purpose of this study is to describe a large retrospective cohort of patients with home mold associated HP.

**Methods:**

HP patients were identified retrospectively from our single center interstitial lung disease (ILD) database between 2011–2019. Patients with a moderate, high, or definite confidence diagnosis of HP were included. The presence of residential mold exposure was confirmed by medical chart review by a pulmonologist trained in exposure assessment.

**Results:**

Home mold exposure was identified as the culprit antigen in 54 of 231 hypersensitivity pneumonitis patients. An invasive procedure (bronchoalveolar lavage, transbronchial biopsy, and/or surgical lung biopsy) was performed to confirm the diagnosis of HP in 85.7% of the cohort. Home mold was principally caused by chronic and/or recurring water intrusion. The most common locations of mold within the home included the bathroom, bedroom, and air conditioning unit. Transplant free survival was 97.7 months, which did not differ from the 50 HP patients in our cohort with HP associated with a mold exposure outside the home or patients in our cohort with HP associated with avian antigen exposure. Of the 41 patients who removed the exposure, 5 (12.2%) had a > 10% improvement in FVC, including 4 with fibrotic HP.

**Conclusion:**

Our study is the largest to report an association between HP and home mold exposure and is the first report from the region of north Texas. As climate change continues to disrupt weather patterns causing storms and flooding in certain areas, clinicians should remain alert to the presence of mold and its potential contribution to development of HP.

## Introduction

Home mold exposure is an important yet underrecognized cause of hypersensitivity pneumonitis (HP). It is known that identification of an inciting antigen is an important first step in the diagnosis of HP, and exposure cessation has prognostic value [1–4]. As such, home mold remediation may play role in improving clinical outcomes. Nonetheless, clinicians continue to overlook mold as a cause of HP due to limited published reports characterizing the association [5–7]. Skeptics have also cited the high prevalence of home mold exposure relative to the low prevalence of HP as well as the lack of accurate antibody testing to confirm sensitization. A large cohort of HP patients with home mold exposure is necessary to strengthen this association.

Concerted efforts have been made to establish a standardized exposure questionnaire including a chronic hypersensitivity pneumonitis survey developed by Barnes et al. using a Delphi method [8]. However, exposure questionnaires to assess for mold within the home are limited by the paucity of data describing specific locations and causes of home mold exposure in HP patients and by variation in the prevalence of home mold based on climate and geography. In addition, the prevalence of home mold may change over time due to climate change and inadequate maintenance of older homes [9,10,11]. This study describes the clinical characteristics and exposure history of a large case series of patients in north Texas who developed mold associated hypersensitivity pneumonitis.

## Methods

We retrospectively identified HP patients evaluated between 2011–2019 from the University of Texas Southwestern Medical Center (UTSW) in Dallas, TX. This study was conducted in accordance with the amended Declaration of Helsinki and was approved by the UTSW Institutional Review Board (STU-2019-0913). Consent was not required due to the inability to obtain consent practically in the retrospective study and the use of de-identified data only. Ethnicity was self-reported by study subjects in the medical record, and subjects were not stratified or analyzed on the basis of confounding variables such as socioeconomic status or nutrition; this information was not available in the medical record and was not included. Data was collected between January 2, 2023–May 3, 2023. Identifying data was not recorded or accessed during or after data collection. To arrive at the diagnosis of HP, HRCT pattern, bronchoalveolar lavage (BAL) lymphocyte percentage, transbronchial biopsy (TBBx) results, and surgical lung biopsy results were evaluated in a multidisciplinary discussion. BAL lymphocyte percentage > 30% was considered supportive of a diagnosis of HP [1]. Patients who had a multidisciplinary diagnosis of moderate, high, or definite confidence of HP by current guidelines due to mold exposure were included [1].

Exposure to potential causative agents was assessed during clinic visits using a template of questions derived from recent published guidelines and asked by the provider to the patient during the initial clinic visit (Supplementary Data 1) [8]. The exposure questionnaire evaluated home environment for water damage or damp conditions, avian antigens, and hobbies and occupations associated with the development of HP. Clinicians noted in the chart whether a formal professional home inspection had been conducted and briefly summarized the findings in the clinic notes. Each exposure was reviewed by an occupational lung disease specialist (CSG) to ensure that the antigen was visible, sufficient in quantity to induce an immunologic response, persistent, and preceded the development of HP [1]. Mold was considered to be removed if any porous material with mold was fully removed from the home and the cause of the water damage was addressed with no residual leak or if the patient moved from the affected home to one without known water damage or mold. Information regarding specific mold species or concentrations are not available.

Clinical data extracted from the medical record included age, gender, smoking history, potential fibrogenic antigen exposure, pulmonary function testing (PFTs), GAP score, date of death or lung transplant, hypersensitivity pneumonitis panel where available, bronchoalveolar lavage cell count and differential, histopathologic interpretation of the transbronchial biopsy and surgical lung biopsy, and date of death or lung transplant [12,13]. Characteristics of home mold exposure recorded included the location of the exposure, duration of the exposure, date of exposure removal and remediation efforts.

### Statistical analysis

Means and standard deviations were used to express continuous variables, while Student’s t test or Wilcoxon signed rank sum test were used to compare them. Counts and percentages were used to express categorical variables, and Chi-squared test or Fisher’s exact test were used to compare them. Univariable and multivariable logistic regression were performed to evaluate association with transplant-free survival. Statistical analyses were performed using MedCalc Statistical Software version 19.2.6 (MedCalc Software bv Ostend, Belgium; https://www.medcalc.org; 2020).

## Results

**Fig 1:**
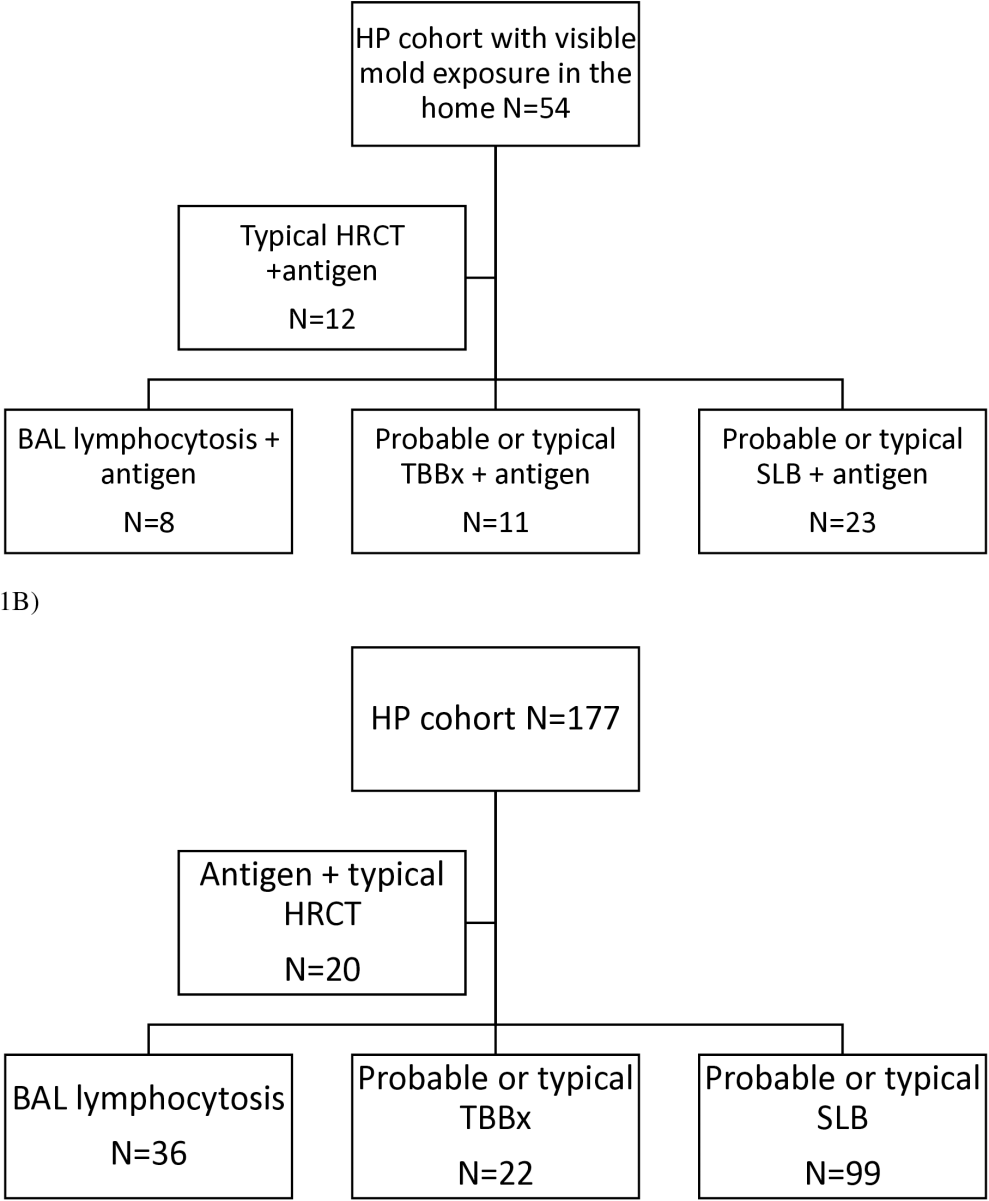
Diagnostic evaluation of HP. A) Home mold associated HP cohort B) HP cohort not associated with home mold.

Our retrospective ILD cohort at UTSW contains 231 patients with a moderate, high, or definite confidence diagnosis of HP. The basis of the diagnosis in each patient is represented in Fig 1. The diagnostic confidence of HP was assigned based on current ATS criteria [2]. Of the 231 patients in the cohort, 54 (23.4%) had HP associated with home mold exposure.

Among the patients with home mold exposure, 48 (89.9%) had fibrotic HP [Table 1]. Mean age at ILD diagnosis was 62.9 years, and 22 (40.7%) were male. The majority were non-Hispanic white. The group had a mean FVC 70.9% + /−18.3% and DLCO 50.9% + /−16.2%. Twenty-two patients (40.7%) required supplemental oxygen. Thirty-one patients (57.4%) had a typical HP pattern HRCT, 6 (11.1%) had compatible with HP HRCT, and 17 (31.5%) had an indeterminate for HP HRCT. Forty-two of 54 patients (77.8%) patients underwent an invasive procedure for diagnosis. For the 12 patients (22.2%) who did not undergo an invasive procedure, the basis of diagnosis was a typical HRCT for HP and identification of antigen exposure by history. Thirty-six patients (66.7%) received pharmacologic treatment with mycophenolate (N = 18), azathioprine (N = 5), prednisone (N = 35), and/or nintedanib (N = 5). Seven (13%) patients died and 5 (9.3%) were transplanted. Median transplant-free survival was 97 months.

**Table 1:** Demographics of cohort.

	HP with home mold exposure cohort (N = 54)	Non home mold HP cohort (N = 177)
Mean age at ILD diagnosis (SD)	62.9 (11.3)	63.4 (11.0)
Male, No. (%)	22 (40.7)	87 (49.2)
Ethnicity, No. (%)		
Non-Hispanic White	43 (79.6)	149 (84.2)
Black	6 (11.1)	5 (2.8)
Hispanic or Latino	2 (3.7)	11 (6.2)
Asian	2 (3.7)	6 (3.4)
Unknown	1 (1.9)	6 (3.4)
Ever smoker, N (%)	22 (40.7)	83 (46.9)
Mold identified in the home, No. (%)	54 (100)	0 (0)
Mold identified outside the home, No. (%)	0	50 (28.2)
Avian antigen	0	97 (54.8)
Other antigen	0	15 (8.5)
Unidentified antigen	0	28 (15.8)
Professional mold inspection	20 (37.0)	0 (0)
Baseline lung function, mean (SD), N		
FVC % predicted	70.1 (18.6), 53	67.0 (18.7), 174
DLCO % predicted	50.9 (16.2), 51	50.5 (18.0), 169
HRCT available for scoring	54 (100)	177 (100)
Typical HP	31 (57.4)	116 (65.5)
Compatible HP	6 (11.1)	19 (10.7)
Indeterminate HP	17 (31.5)	42 (23.7)
HRCT with air trapping or centrilobular nodules	37 (68.5)	131 (74.0)
Fibrotic HP	48 (89.9)	149 (84.2)
Invasive procedure performed	42 (77.8)	156 (88.1)
Surgical biopsy	28 (51.9)	111 (62.7)
TBBx	23 (42.6)	69 (39.0)
BAL	20 (37.0)	60 (33.9)
Confidence HP diagnosis by American Thoracic Society Criteria[2]		
Moderate	26 (48.1)	66 (37.3)
High	5 (9.3)	32 (18.1)
Definite	23 (42.6)	79 (44.6)
Pharmacologic treatment for > 6 months	36 (66.7)	121 (68.4)
Mycophenolate	18 (33.3)	71 (40.1)
Azathioprine	5 (9.3)	26 (14.7)
Prednisone	35 (64.8)	116 (65.5)
Nintedanib	5 (9.3)	7 (4.0)
Outcomes		
Death	7 (13.0)	29 (16.4)
Transplant	5 (9.3)	31 (17.5)
Median transplant-free survival in months	97.7	51.0

**By current guidelines, patients with an SLB indeterminate for HP can qualify for a diagnosis of at least moderate confidence HP based on antigen exposure, characteristics of the HRCT, and BAL lymphocytosis[2]

Among those with mold exposure in the home, 20 (37%) underwent professional mold inspection by an industrial hygienist, and in the remainder visible mold was confirmed by the patient, a family member, or a contractor. Serum precipitins were not available for our cohort, as they are not routinely used at our institution for patients with a clear exposure due to poor negative predictive value in that setting [9].

Among patients with mold exposure in their place of residence, the majority of patients (81.5%) lived in a house, while 8 patients (14.8%) lived in an apartment [Table 2]. The most common locations of mold within the home included the bathroom (16.7%), bedroom (18.5%), and central air conditioning unit (22.2%). Eighteen patients (33.3%) had more than one area with mold in the home, and 8 patients (14.5%) had more than 2 areas with mold. Sources of water intrusion most commonly included a pipe leak or burst (33.3%), roof leak (14.1%), and leak under a pier and beam foundation (11.1%). Nine patients (16.7%) had more than one source of water intrusion. Five patients (9.3%) reported mold development after a single episode of a pipe burst which was not remediated, but for 49 patients (90.7%) the leak was due to a recurrent or chronic issue, such as a chronically leaking pipe or a basement with recurrent flooding episodes.

**Table 2:** Characteristics of exposure.

	HP cohort (N = 54)
Type of residence with mold exposure	54 (100)
House	44 (81.5)
Apartment/condominium	9 (16.7
RV	1 (1.9)
Location of visible mold in the home*	
Bathroom dry wall	9 (16.7)
Laundry Room	4 (7.4)
Bedroom	10 (18.5)
Living area	5 (9.3)
Basement	3 (5.6)
Kitchen	3 (5.6)
Air conditioning unit	13 (24.0)
Location of mold not recorded	8 (14.8)
Type of water intrusion**	
Roof leak	8 (14.8)
Pipe leak or burst	18 (33.3)
Flood	4 (7.4)
Water heater leak	2 (3.7)
Washing machine leak	3 (5.6)
Toilet leak	4 (7.4)
Leak from windows	3 (5.6)
Slab leak	1 (1.9)
Leak under pier and beam foundation	6 (11.1)
Type of water intrusion not recorded	9 (16.7)
Duration of water intrusion	
Single episode	5 (9.3)
Recurrent and/or persistent	49 (90.7)
Removed exposure	41 (75.9)
Moved	21 (38.9)
Professional remediation	20 (37.0)
Median time from ILD diagnosis to removal in months	11.6
Response to exposure removal, N = 41	
> 10% improvement in FVC	5 (12.2)
No significant change	24 (58.5)
> 5% decline in FVC	0 (0)
Unable to assess***	12 (29.3)

* 18 patients (33.3%) had more than one area with mold in the home, and 8 patients (14.5%) had more than 2 areas with mold.

**9 patients (16.7%) had more than one type of water intrusion

***Unable to assess for improvement in FVC after exposure removal in patients who did not have PFTs before removal or after removal or do not have a clear removal date

Forty-one patients (75.9%) removed the home mold exposure, and of these 20 patients (37.0%) had a professional mold remediation while 21 (38.9%) moved from the affected residence. The median time from ILD diagnosis to mold remediation or moving from the residence was 11.6 months, and all patients had follow up for at least 3 months after diagnosis. Of those who removed the exposure, 5 (12.2%) had a > 10% improvement in FVC % predicted on follow up PFTs performed between 3 and 4 months after exposure removal, 24 (58.5%) had no significant change, 0 (0%) had > 10% decline in FVC % predicted, and 12 (29.3%) had insufficient data recorded to determine improvement.

Of the 5 patients who improved by > 10% predicted FVC within 3–4 months of antigen removal, 4 (80%) were fibrotic and 1 (20%) was nonfibrotic; 3 (60%) had undergone mold remediation and 2 (40%) had moved. In the patient with nonfibrotic HP, HRCT revealed resolution of ground glass opacities following exposure removal. Two fibrotic HP patients who improved with exposure did not have follow-up HRCT and two had an HRCT following exposure removal that was unchanged. All 5 patients who had > 10% improvement in FVC following exposure removal maintained their improved FVC at 1 year from exposure removal [Table 3]. Three of these patients were lost to follow-up within 18 months. Two patients with follow-up duration of longer than 2 years eventually experienced a decline in FVC by 10% predicted between 3 and 5 years following exposure removal; these patients both had fibrotic HP.

**Table 3:** Characteristics of patients who had > 10% improvement in FVC on PFTs done 3-4 months following exposure removal.

Patient	Fibrotic vs nonfibrotic HP	Clinical course
Patient 1	Fibrotic	Maintained improvement in FVC for 3 years, then declined by 10% in the next 2 years; alive at censor date; follow up HRCT was unchanged
Patient 2	Nonfibrotic	Maintained improvement in FVC for 18 months, then lost to follow up; alive at censor date; follow up HRCT demonstrated improvement in ground glass opacities
Patient 3	Fibrotic	Maintained improvement in FVC for 3 years, then declined by 10% over the subsequent 18 months; deceased; follow up HRCT was unchanged
Patient 4	Fibrotic	Maintained improvement in FVC for 14 months, then lost to follow up; alive at censor date; follow up HRCT not performed.
Patient 5	Fibrotic	Maintained improvement in FVC for 1 year, then lost to follow up; alive at censor date; follow up HRCT not performed.

Our HP cohort contained 177 patients that did not have home mold exposure. Of these, 87 (49.2%) were male, the majority were non-Hispanic white, and the group had moderately impaired lung function at baseline [Table 1]. Twenty-eight patients (15.8%) did not have an identified antigen, 50 (28.2%) had mold identified outside the home and 97 (54.8%) had avian antigen. The majority of patients (84.2%) had fibrotic HP.

When HP patients with home mold associated HP were compared to HP patients with mold exposure outside the home, there was no difference in transplant-free survival between groups in a univariable (HR 0.60, 95% CI 0.29–1.21, p = 0.16) or multivariable model adjusted for GAP score (HR 0.79, 95% CI 0.35–1.70, p = 0.54). The incidence of fibrotic HP was not different between groups (89.9% vs 84.2%, p = 0.41). Mold exposure outside the home was most commonly related to farming (N = 16) and saunas (N = 8). There was similarly no difference in transplant-free survival when patients with home mold associated HP were compared to patients with avian antigen exposure (data not shown).

## Discussion

In this case series, we describe 54 patients with hypersensitivity pneumonitis associated with home mold exposure, accounting for 23% of our entire HP cohort. The most common locations of mold within the home included the bathroom, bedroom, and central air conditioning unit. Sources of water intrusion most commonly included a pipe leak or burst, roof leak, and leak under a pier and beam foundation. The majority of types of water intrusion leading to mold growth were chronic and/or recurring. Transplant free survival in this cohort was 97.7 months, which did not differ from HP patients with a mold exposure outside the home. Our study further strengthens the association between home mold exposure and HP.

HP from residential mold exposure has been previously described. In Japan, summer-type hypersensitivity pneumonitis attributed to mold contamination principally by Trichosporon species is widely reported [14–16]. The evidence outside that region is more limited. Individual case reports provided initial clues about the potential deleterious effects of home mold exposure and often detailed the extent of the mold exposure. As an example, a 50 year old woman developed HP in a suburban residence with a history of a major water leak that only resolved after moving to a new home A number of species were grown from samples obtained from basement sump pump water, fiberglass and the carpet, including*Aureobasidium pullulans*, *Curvularia* species, and *Humicola* species [17]. Another report described a case of fatal HP in a 37 year old male who rapidly progressed after self-renovating his water damaged home; *Fusarium vasinfectum* was cultured from home samples [5]. While such cases reports are helpful in guiding clinicians, very few of these reports exist in the literature.

Larger series of patients have been more recently described [18–21]. A scoping review of several hundred HP studies, that included a range of studies from prospective ILD registries to case series and case reports, noted mold exposure accounted for 17% of HP cases [22]. A closer look at these studies reveal that specific information regarding the mold exposure is lacking. Seventy eight (43.1%) of 261 hypersensitivity cases were attributed to environmental mold in a single center study in the Southeastern United States. While the authors postulated that high humidity conditions in a region near the Atlantic Ocean contributed to mold growth, specific description of the location and extent of mold in the series was absent [18]. (Gu 2020). Among 202 HP patients in a Belgian study, 40 cases were attributed to mold, with 22 of those having fibrotic HP [19]. The authors report that individuals with avian exposures had better outcomes as compared to mold exposed patients. Nonetheless, little information regarding the mold exposure itself is provided.

While other studies have used a combination of radiologic, histopathologic and exposure data to establish a diagnosis of HP, our study explicitly categorized cases according to more recently established ATS criteria [1,2]. Our study also provides more specific descriptions of the source, extent and duration of water damage as compared to other studies. Additionally, recent CHEST guidelines have emphasized the importance of classifying the degree of certainty that an antigen is causative; an inciting antigen is categorized as identified, indeterminate and unidentified. In our cohort, a trained occupational ILD pulmonologist confirmed the presence of significant mold exposure.

There are several weaknesses which should be acknowledged. Follow up length of time is limited for some patients, and follow up HRCTs are not available for some patients where improvement of FVC was suggested.

Additional limitations include potential weaknesses in exposure assessment. Home evaluations by certified specialist were not universally conducted. Only the clinician’s brief summary of the formal reports was available as part of the clinical record.

At our center serologic testing to document individual patient sensitization is not performed. IgG methods to establish the presence of exposure, while valuable in certain scenarios, are often insufficient [1,9]. Commercial serum IgG testing through ELISA or precipitin testing to document individual patient sensitization has at best modest sensitivity and specificity due to the lack of standardization of methodology and quality. A recent study suggested potential misalignment between serum IgG testing and the specific mold found in the patient’s environment [23]. The evaluation of patient serum for specific antibodies to environmental samples collected from a patient’s home has been proposed. While seemingly a more personalized approach, such a strategy is limited by possible failure to identify the right antigen or source as well as practical limitations as such as time, cost and access to the environment [24].

Environmental sampling for molds has been proposed in the diagnostic assessment of HP [9,23]. Industrial hygiene evaluations and environmental sampling was conducted in some patients; however, specific details regarding quantitative sampling and the specific species identified are also lacking in our study Standardized sampling methods as well as reference ranges have not been established for mold [23]. Surface samples may identify specific species but do not quantify airborne levels. In addition, culture media may not allow growth of certain suspected organisms [23]. Ultimately, visual inspection is often superior to quantitative air sampling, and a history of water intrusion provides strong supporting evidence of the presence of conditions ideal mold overgrowth [9,23].

While summer type HP in Japan is a well-recognized entity, clinicians outside the region have continued to overlook home mold exposure as a potential cause of HP. While the type of antigen leading to HP (avian or mold) has not been consistently associated with differential survival [22], the importance of identification of antigen in the diagnosis of HP has been demonstrated in prior studies [25,26]. HP diagnostic confidence increases when antigen is identified, which may reduce the number of diagnostic procedures [1,2]. Furthermore, exposure removal may slow disease progression in HP or even result in improvement, albeit less likely in fibrotic patients but still possible [3,26]. By characterizing home mold exposures, our study provides valuable data for clinicians taking an exposure history and supporting the process of mold remediation.

In summary, we describe a large case series of mold-related HP in north Texas and provide detailed description of the sites of home mold exposure. These results support that home mold may be an important and modifiable cause of HP. As climate change continues to disrupt weather patterns causing storms and flooding in certain areas, clinicians should remain alert to the presence of mold and its potential contribution to development of HP.

## Supporting information

S1 File Home mold case series data deidentified FTC. (XLSX)
